# The surgical management of women with ovarian cancer in the south west of England

**DOI:** 10.1054/bjoc.2001.2196

**Published:** 2001-12

**Authors:** A Olaitan, J Weeks, A Mocroft, J Smith, K Howe, J Murdoch

**Affiliations:** Department of Gynaecological Oncology, St Michael's Hospital, Southwell Street, Bristol, BS2 8EG; Regional Cancer Organisation, Avon Health Authority; Department of Primary Care & Population Sciences, Royal Free & University College Medical School, Royal Free Campus, Rowland Hill Street, London, NW3 2PF, UK

**Keywords:** ovarian cancer, surgery, cytoreduction, gynaecological oncologist

## Abstract

The surgical management of epithelial ovarian cancer in the South West of England was studied in the two years 1997–1998 in order to determine the factors that influence the outcome of surgery and to provide a baseline from which to assess the effect of centralisation of cancer services. All hospitals in the South West region of England participating in the Regional Cancer Organisation's longitudinal study of outcomes in gynaecological malignancies are included. Six hundred and eighty-two patients with epithelial ovarian cancer were registered with the RCO in the two-year study period. Five hundred and ninety-five women were offered primary cytoreductive surgery of which 438 were said to be optimally cytoreduced. Applying multivariate models to analyse the outcome of surgery, older patients (OR = 0.82 per 5-year increase in age, *P* = 0.0003), patients treated in hospitals managing fewer than ten cases of ovarian cancer per year (OR = 1.92, *P* = 0.02) and patients with FIGO stage 3 (OR = 0.02, *P* < 0.0001) or 4 (OR = 0.002, *P *< 0.0001) disease were less likely to be optimally cytoreduced. Gynaecological oncologists were 2.06 times more likely to attain optimal cytoreduction when compared to general gynaecologists and this was statistically significant (*P* = 0.01). The results from this study support the argument that limiting surgery for ovarian malignancy to specialised surgeons improves the extent of cytoreductive surgery. © 2001 Cancer Research Campaign http://www.bjcancer.com


*British Journal of Cancer* (2001) **85**(12), 1824–1830
© 2001 Cancer Research Campaign
doi: 10.1054/ bjoc.2001.2196, available online at http://www.idealibrary.com on IDEAL

http://www.bjcancer.com

### The surgical management of women with ovarian cancer in the south west of England

**A Olaitan[1], J Weeks[2], A Mocroft[3], J Smith[1], K Howe[1], and J Murdoch[1]**

[1]Department of Gynaecological Oncology, St Michael's Hospital, Southwell Street, Bristol, BS2, 8EG; [2]Regional Cancer Organisation, Avon Health Authority; [3]Department of Primary Care & Population Sciences, Royal Free & University College Medical School, Royal Free Campus, Rowland Hill Street, London NW3 2PF, UK

**Summary** The surgical management of epithelial ovarian cancer in the South West of England was studied in the two years 1997–1998 in order to determine the factors that influence the outcome of surgery and to provide a baseline from which to assess the effect of centralisation of cancer services. All hospitals in the South West region of England participating in the Regional Cancer Organisation's longitudinal study of outcomes in gynaecological malignancies are included. Six hundred and eighty-two patients with epithelial ovarian cancer were registered with the RCO in the two-year study period. Five hundred and ninety-five women were offered primary cytoreductive surgery of which 438 were said to be optimally cytoreduced. Applying multivariate models to analyse the outcome of surgery, older patients (OR = 0.82 per 5-year increase in age, $P = 0.0003$), patients treated in hospitals managing fewer than ten cases of ovarian cancer per year (OR = 1.92, $P = 0.02$) and patients with FIGO stage 3 (OR = 0.02, $P < 0.0001$) or 4 (OR = 0.002, $P < 0.0001$) disease were less likely to be optimally cytoreduced. Gynaecological oncologists were 2.06 times more likely to attain optimal cytoreduction when compared to general gynaecologists and this was statistically significant ($P = 0.01$). The results from this study support the argument that limiting surgery for ovarian malignancy to specialised surgeons improves the extent of cytoreductive surgery. © 2001 Cancer Research Campaign http://www.bjcancer.com

**Keywords**: ovarian cancer; surgery; cytoreduction; gynaecological oncologist

### INTRODUCTION

The publication of the Calman-Hine Report (Department of Health, 1995) on the commissioning of cancer services has focused attention on the organisation of services for gynaecological cancer. Gynaecological cancers are, by definition, rare cancers and it has been advocated that the centralisation of services will lead to improved quality of care. This assertion has been supported by several recent publications (Jackson et al, 1997), particularly relating to ovarian cancer (Junoer et al, 1994; Kehoe et al, 1994). Ovarian cancer, the commonest gynaecological cancer, has a poor outcome, with a five-year survival of 30–40% (Nguyen et al, 1993). This has been attributed, in part, to late presentation of disease, with the majority of cases having extra-ovarian spread at diagnosis (Nguyen et al, 1996). The principles employed in the treatment of epithelial ovarian cancer are consensus rather than research-based and involve surgical resection of the disease, followed by chemotherapy. Prescribed optimal cytoreductive surgery, which involves reducing the disease to less than 2 cm, can be particularly challenging in cases of advanced ovarian cancer. Previous publications have demonstrated that Gynaecological Oncological surgeons achieve better outcomes in terms of smaller residual disease after surgery (Junor et al, 1994) and improved survival (Kehoe et al, 1994; Junor et al, 1999) than their general gynaecological or surgical colleagues.

The South West region of England is a large geographical region, stretching 220 miles from Gloucester in the north of the region to Penzance at its southernmost tip. The region is served by five cancer centres: Three Counties Centre to the north of the region which serves Cheltenham, Gloucester and Hereford hospitals; Avon and Somerset cancer centre based in Bristol; Exeter and Plymouth cancer centres in the south west of the region and Dorset in the south east. Wiltshire became a part of Avon and Somerset cancer centre at the end of 1998 (See Appendix 1). The current organisation of gynaecological cancer services in the South West of England is similar to the rest of the country. The regional cancer centre receives primary referral from its catchment population and tertiary referrals of more complicated or relapsed cases from associated cancer units and district hospitals. Most ovarian cancer cases receive their primary treatment in their local hospital and are referred centrally if they require further surgery or chemotherapy. This arrangement may change in the near future following the implementation of the recently published National Health Service Executive document 'Improving Outcomes in Gynaecological Cancer' (NHS Executive, 1999). In this document, it has been advocated that all women with ovarian cancer be referred to recognised cancer centres so that they can receive optimal management for their disease, with multidisciplinary input into the planning of their management, expert surgery and chemotherapy from dedicated gynaecological surgeons and oncologists.

There are particular challenges to developing this model of care in a region with a widespread rural population. We therefore decided to study the surgical management of epithelial ovarian cancer in the South West of England in the two years 1997–1998

*Received 4 September 2000*
*Revised 17 September 2001*
*Accepted 3 October 2001*

*Correspondence to:* A Olaitan

On behalf of: South and West Tumour Panel for Gynaecological Cancer R Anderson, P Bliss, I Boyd, T Clarke, R Counsell, J Cullimore, F Daniel, A Falconer, E Gilby, J Giles, J Graham, L Hirschowitz, A Hong, N Johnson, V Laurence, R Marshall, JB Murdoch, J Orford, JN Renninson, J Richardson, G Swingler prior to the re-organisation of gynaecological cancer services, looking particularly at factors influencing the completeness of surgery at each stage of disease. This study, as well as providing a base-line against which future performance can be measured, enables us to estimate the increase in work load and the resource implications for the regional cancer centre if centralisation of ovarian cancer management is fully implemented.

### MATERIALS AND METHODS

The South and West Regional Cancer Organisation (RCO) Gynaecology Tumour Panel is constituted of a multidisciplinary team of experts involved in the management of gynaecological malignancies. In 1997, the RCO initiated a prospective longitudinal study of the management of gynaecological cancer to establish current patterns of care and facilitate development of services in the region (Jackson et al, 1997). A one-page minimum data-set for each major gynaecological cancer site was designed (Figure 1). After obtaining approval from all units managing gynaecological malignancies, copies of the minimum data set pro forma were circulated along with guidance notes that included outlines of American Society of Anesthesiologists (ASA) grading, FIGO staging, and histopathology information required. Treatment policies detailing accepted pre-operative and operative management of epithelial ovarian cancer were produced and circulated by the RCO to all participating hospitals (Appendix 1). To ensure uniform histopathological reporting, the RCO also circulated guidelines for participating histopathologists (Appendix 2). The clinician managing the case is responsible for filling the form and for returning a copy to the RCO. The data are then entered on a central Microsoft Access™ database managed by one of the co-authors (JW). Complete data on surgical procedures are available for 1997 and 1998.

Data for all ovarian cancer cases registered with the RCO from January 1997 to December 1998 were obtained from the RCO database. Central validation by the RCO had previously confirmed registration rates of close to 100% for all hospitals participating in 1997 (Regional Cancer Organisation, 1999). To ascertain completeness of the data and to identify outstanding cases not registered with the RCO, the histopathology departments of participating hospitals were approached for lists of patients with a histological diagnosis of ovarian cancer during the study period. Any cases thus identified were checked with the appropriate clinician, yielding some additional registrations.

The total number and FIGO stage of epithelial ovarian cancer cases managed by each contributing hospital per year was calculated. The accuracy of the FIGO stage was examined by comparing the ascribed FIGO stage with the clinico-pathological data supplied on the RCO form. These details were checked against the specialisation of the surgeon responsible for the case. Cases were re-staged by one of the authors (AO) if there was a mismatch between ascribed FIGO stage and the clinico-pathological data available. Where no FIGO stage was stated, patients were allocated a stage where there was sufficient staging information supplied on the RCO form. Sub-stages were ascribed or confirmed where this information was available. Cases were left without a clinical stage where there was insufficient clinico-pathological information available. Cases were also left unstaged where there was insufficient information to confirm the original FIGO stage allocated. The amended FIGO staging was applied in all subsequent analysis.

All cases undergoing primary surgery were classified according to extent of surgery per stage of disease. Patients with less than 2 cm residual disease at the end of surgery were classified as receiving optimal cytoreductive surgery while those with greater than 2 cm residual disease were classified as receiving sub-optimal surgery.

### Statistical methods

Univariate and multivariate logistic regression analyses were applied to determine the factors related to both the decision to offer surgery and on the outcome of surgery. The factors investigated included age, ASA grade, stage of disease, grade and specialisation of surgeon, consultant supervision, and the number of ovarian cancer operations per contributing hospital per year (classified as 10 or fewer cases of ovarian cancer per year or greater than 10 cases per year).

The operating surgeons were classified as general surgeons, general gynaecologists and gynaecological oncologists according to the information supplied on the RCO form. A gynaecological oncologist was defined as an RCOG-recognised sub-speciality trained individual (of which there is one in the region) or the lead Consultant for gynaecological oncology within a cancer centre. In addition, lead clinicians for gynaecological cancer within teaching or district hospitals with a large caseload (15 or more cases of ovarian cancer per surgeon per year) were included in this definition, giving a total of nine gynaecological oncologists within the region.

### RESULTS

A total of 820 ovarian cancer cases were registered with the RCO in the study period 1997–1998 from 20 NHS and eight private hospitals. Of these, 36 were non-epithelial tumours. Non-epithelial cancers are not considered in this study and have been excluded from all further analyses. A further eight cases were excluded as they had secondary ovarian tumours from a non-gynaecological primary. Other exclusions included 15 patients with sychronous primaries, the second tumour being non-ovarian and 21 patients who were registered with recurrent ovarian cancer. Of the 740 remaining patients, 58 were registered by hospitals outside the south west region where we were unable to crosscheck the number of ovarian cancer cases with the histopathology department. These patients were therefore also excluded, leaving a total of 682 cases registered with the RCO with a verified diagnosis of primary epithelial ovarian cancer. This number included 129 cases of borderline epithelial tumours. The median age of these patients was 62 years (range 18–94). Figure 2 illustrates the total number of cases registered by each hospital per year. Some hospitals only started to register gynaecological cancer cases with the RCO in 1998 and this is reflected in the relatively small number of cases registered. A total of 94 surgeons, including seven general surgeons and one breast surgeon managed these cases. The median number of cases managed per surgeon over the two-year study period was four (range 1–72). Figure 3 illustrates the number of ovarian cancer cases undertaken by the lead clinician for gynaecological cancer services in each contributing hospital in the two year study period. The Dorset hospitals (see Appendix 1) are not included in this figure as they only started to register cases with the RCO in 1998.

Eighty-nine patients were re-staged because the clinico-pathological information given on the RCO form did not match the FIGO stage ascribed while there were 132 cases where there was insufficient data to confirm or assign a stage, making a total of 221

*© 2001 Cancer Research Campaign*

### SOUTH AND WEST REGIONAL CANCER ORGANISATION

**Figure 1** Regional Cancer Organisation Registration Pro forma

*© 2001 Cancer Research Campaign*

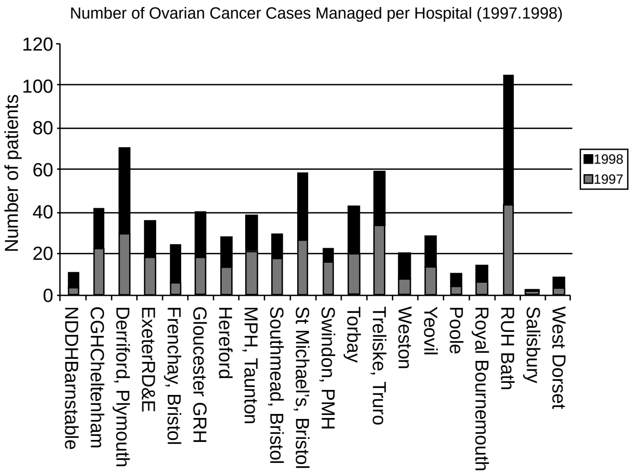

**Figure 2** Number of ovarian cancer cases managed per hospital 1997–1998

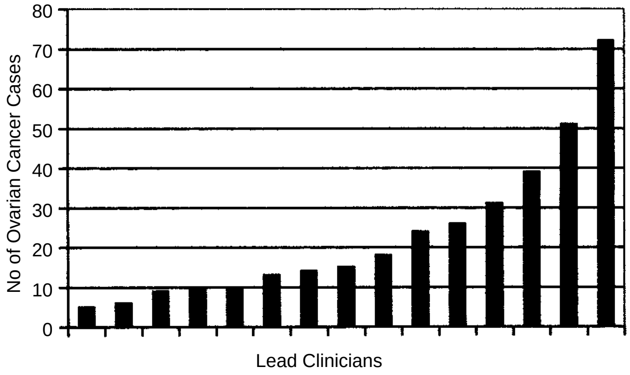

**Figure 3** Number of ovarian cancer cases/lead clinician in two-year study period

(32.5%) unsatisfactorily staged cases. Table 1 describes the re-staging action taken classified according to the specialisation of the surgeon managing the case. Staging was accurate in 81.6% of cases managed by gynaecological oncologists, 62.8% of cases managed by general gynaecologists and 32.1% of cases managed by general surgeons.

Five hundred and ninety-five patients (87.2%) were managed surgically while 69 (9.9%) had laparoscopy and biopsy only. In 438 (81.2%) of the surgical cases, the Consultant was the primary surgeon. In 14 cases, the grade of the primary surgeon was not indicated. The remaining 98 were operated on by surgeons in non-Consultant grades, specialist registrars in the majority of cases. Sixty-two (63.0%) of these procedures were undertaken under Consultant supervision. In all 545 (92%) of procedures were performed by consultants or under consultant supervision. The surgeons undertaking ovarian cancer surgery without supervision included specialist registrars in year 3 (three cases), year 4 (three cases), year 5 (12 cases) and nine cases undertaken by a variety of non-consultant grade surgeons including staff grade doctors.

The amended FIGO stage at presentation of patients who underwent definitive surgery and those who did not is described in Table 2. Table 3 shows the ASA grade of patients undergoing surgery compared with those managed conservatively.

Four hundred and thirty-eight (73.8%) were described as having less than 2 cm of residual disease after surgery while 119 women had more than 2 cm of residual disease. Table 4 describes the FIGO stage at presentation of these patients and the cytoreductive rates tabulated against the operating surgeon's specialisation.

Applying univariate analysis to determine the factors influencing the decision to undertake surgery revealed that patients were less likely to be offered surgery if they were of older age (odds ratio (OR) per 5-year increase in age = 0.89, $P = 0.005$), in ASA grade 3 or 4 (OR 0.30, $P < 0.0001$) and had FIGO stage 4 disease at presentation (OR=0.10, $P < 0.0001$). In multivariate analyses, age was no longer significant, but patients in ASA grade 3 or 4 remained significantly less likely to be offered surgery (OR 0.3, $P < 0.0001$), as were patients in FIGO stage 4 (OR 0.10, $P = 0.0001$). In addition, in multivariate analyses, there was no significant difference in the likelihood of undergoing surgery by the

**Table 2** FIGO stage of ovarian cancer at presentation comparing patients who underwent primary surgery with those who did not

| FIGO Stage | Total no. of cases (%) | Total no. with surgery (%) | Total no. no surgery (%) |
|---|---|---|---|
| I | 156 (22.9) | 155 (22.7) | 1 (1.2) |
| II | 51 (07.5) | 51 (7.4) | 0 (0.0) |
| III | 318 (46.6) | 272 (39.9) | 46 (6.7) |
| IV | 24 (3.5) | 13 (1.9) | 11 (1.6) |
| Unstated | 133 (19.5) | 104 (15.2) | 29 (4.3) |
| Total | 682 (100) | 595 (87.2) | 87 (12.8) |

**Table 3** ASA grades of patients undergoing surgery compared with those who had no surgery

| ASA grade | Total no. of cases (%) | Total no with surgery (%) | Total no with no surgery (%) |
|---|---|---|---|
| 1 | 293 (43.0) | 277 (40.6) | 16 (2.3) |
| 2 | 204 (29.9) | 179 (26.2) | 25 (3.7) |
| 3 | 104 (15.2) | 78 (11.4) | 26 (3.8) |
| 4 | 8 (1.2) | 4 (0.6) | 4 (0.6) |
| not stated | 73 (10.7) | 57 (8.4) | 16 (2.3) |
| Total | 682 (100) | 595 (87.2) | 87 (12.8) |

**Table 1** Amended stage of ovarian cancer classified according to specialisation of responsible surgeon

| Specialisation | No Change | Down-staged | Up-staged | Staged | Up-substage | Down-substage | No Stage | Total |
|---|---|---|---|---|---|---|---|---|
| Gynae | 243 | 27 | 10 | 19 | 4 | 5 | 79 | 387 (56.7) |
| Gynae-Onc | 199 | 5 | 5 | 5 | 0 | 1 | 29 | 244 (35.8) |
| Surgeon | 9 | 2 | 1 | 0 | 0 | 0 | 16 | 28 (4.1) |
| Not Stated | 10 | 3 | 1 | 1 | 0 | 0 | 8 | 23 (3.4) |
| Total | 461 | 37 | 17 | 25 | 4 | 6 | 132 | 682 |
| (%) | (67.5) | (5.4) | (2.5) | (3.7) | (0.6) | (0.9) | (19.4) | (100) |

© 2001 Cancer Research Campaign 

**Table 4** FIGO stage of patients undergoing optimal cytoreductive surgery classified according to surgeon's specialisation

| FIGO stage | Gynaecologist 341 | | | Gynaecological oncologist 219 | | | Surgeon 20 | | | Unstated 15 | | | Total 595 |
|---|---|---|---|---|---|---|---|---|---|---|---|---|---|
| Total | < 2 cm | > 2 cm | Unst | < 2 cm | >2 cm | Unst. | <2 cm | >2 cm | Unst. | <2 cm | >2 cm | Unst. | |
| I | 94 | 1 | 7 | 46 | 0 | 2 | 1 | 0 | 0 | 1 | 0 | 0 | 152 |
| II | 23 | 1 | 1 | 20 | 0 | 0 | 1 | 0 | 2 | 4 | 0 | 1 | 53 |
| III | 67 | 66 | 7 | 82 | 36 | 6 | 1 | 3 | 1 | 3 | 0 | 1 | 273 |
| IV | 0 | 6 | 2 | 4 | 0 | 0 | 0 | 0 | 0 | 0 | 0 | 0 | 12 |
| Unst. | 62 | 1 | 5 | 20 | 0 | 1 | 10 | 1 | 0 | 2 | 0 | 3 | 105 |
| Total | 246 | 75 | 22 | 172 | 36 | 9 | 13 | 4 | 3 | 10 | 0 | 5 | 595 |
| % | 71.8 | 22.0 | 6.2 | 77.6 | 18.3 | 4.1 | 65.0 | 20.0 | 15.0 | 66.7 | 0.0 | 33.3 | 100 |

Unst = Unstated caseload of the hospital (OR = 1.01, *P* = 0.97) or by the specialisation of the surgeon (OR = 1.33, *P* = 0.32) when comparing gynaecological oncologists with general gynaecologists.

These factors also had an effect on the outcome of surgery. In multivariate models, older patients were less likely to be optimally cytoreduced (OR = 0.82 per 5-year increase in age, *P* = 0.0003). Similarly patients with FIGO stage 3 (OR = 0.02, *P* < 0.0001) or 4 (OR = 0.002, *P* < 0.0001) disease were less likely to be optimally cytoreduced. Patients treated in hospitals managing fewer than 10 cases per year were less likely to be optimally cytoreduced (OR = 1.92, *P* = 0.02) compared to those treated in hospitals managing 10 or more cases per year. ASA grade appeared to have no effect on the extent of surgery. Gynaecological oncologists were significantly more likely to attain optimal cytoreduction when compared to general gynaecologists (OR = 2.06, *P* = 0.01) while general surgeons were less likely to attain optimal cytoreduction although this difference was not statistically significant (OR = 0.30, *P* = 0.18).

### DISCUSSION

The peculiar geography of the South West Region may make centralisation of gynaecological cancer services particularly challenging. The large expanse of rural and sparsely populated land means that there are several hospitals serving relatively small populations. Consequently, the majority of surgeons participating in this survey managed fewer than ten cases of ovarian cancer per year. The median number of cases per surgeon was four and over half of the lead surgeons for cancer services were operating on fewer than ten cases of ovarian cancer per year. The need to maintain a critical volume of work in order to sustain surgical expertise has been extensively discussed (Department of Health 1995; Jackson et al, 1997). Trimbos et al (2000), using surgery for cervical cancer as a model, demonstrated that there is a long learning curve associated with surgery for gynaecological cancer. They demonstrated a reduction in operative blood loss and operating time achieved by the same operating team over a period of thirteen years.

Eighty-nine patients (13%) required re-staging due to discrepancies between the ascribed FIGO stage and the clinico-pathological data supplied on the RCO form. One hundred and thirty-two patients (19.4%) could not be allocated a stage because there was insufficient staging data available on the RCO form. The majority of cases managed by gynaecological oncologists were accurately staged but this proportion was decreased in general gynaecologists and more so for general surgeons where, in the majority of cases, there was insufficient staging information recorded. Accurate staging is of critical importance as the decision about whether to recommend adjuvant therapy is based on this. In addition, analyses of survival data become meaningless if the correct FIGO stage is not established at the outset.

Although the same selection criteria for surgery were apparently applied through all centres, with older, less fit women being less likely to be offered surgery, hospitals that managed more than ten cases of ovarian cancer a year achieved better optimal cytoreduction rates than those managing ten or fewer cases per. In keeping with previous published reports (Junor et al, 1994; Kehoe et al, 1994), sub-specialist gynaecological oncologists achieved significantly better optimal cytoreduction rates than their non-sub-specialist colleagues although the majority of operations were carried out by general gynaecologists. The improved cytoreduction rates achieved by gynaecological oncologists (as defined by this paper) may reflect their higher case volume. There is only one RCOG-recognised subspecialty trained gynaecological oncologist in the region and so the other 'gynaecological oncologists' included in this analysis were defined by their work-load. The more widespread introduction of sub-specialty training in gynaecological oncology will, in future, allow tighter definitions of specialists and consequently, more specific analyses.

Although there are no randomised controlled trials analysing the effect of optimal cytoreductive surgery on median survival, a large number of retrospective studies have documented a favourable prognostic effect on median survival (Hogberg et al, 1993; Marsoni et al, 1990). A meta-analysis of 58 studies showed that maximum cytoreductive surgery produced a small improvement in median survival time (Hunter et al, 1992).

It is of concern that, although this happened infrequently, a number of junior doctors undertook surgery for ovarian cancer without supervision. However, the RCO does not routinely collect data about which cases were undertaken as emergencies and it may be that some of these cases presented acutely and that there was no prior suspicion of cancer. Appropriately, the majority of operations (92%) were undertaken by or supervised by Consultants.

The main drawback of this analysis is the failure to include data on chemotherapy. There is a lag phase of up to six months between surgery and completion of chemotherapy and the data for 1998 are as yet, incomplete. We would aim to include chemotherapy data in our subsequent analysis.

The follow-up period in this study is too short to examine survival, particularly as chemotherapy data are incomplete. However, previous publications (Junor et al, 1999) have shown a survival advantage in patients operated on by gynaecological oncologists over general

 *© 2001 Cancer Research Campaign*

gynaecologists or surgeons. Longitudinal follow-up is on-going and we should be in a position to analyse survival next year with the advantage of prospectively collected data.

### ACKNOWLEDGEMENTS

This analysis would not have been possible without the co-operation of the Consultants from all participating hospitals who worked hard to ensure that the RCO forms were completed and promptly returned.

### REFERENCES

Department of Health, May 1995. A policy framework for commissioning cancer services. Guidance for purchasers and providers of cancer services.
Hogberg T, Carstensen J and Simonsen E (1993) Treatment results and prognostic factors in a population-based study of ovarian cancer. *Gynecol Oncol* **48**: 38–49
Hunter RW, Alexander ND and Soutter WP (1992) Meta-analysis of surgery in advanced ovarian carcinoma: is maximum cytoreductive surgery an independent determinant of prognosis? *Am J Obstet Gynecol* **166**: 504–511
Jackson S, Murdoch J, Howe K et al (1997) The management of cervical carcinoma within the South West region of England. *Br J Obstet Gynaecol* **104**: 140–144
Junor EJ, Hole DJ and Gillis CR (1994) Management of ovarian cancer: referral to a multidisciplinary team matters. *Br J Cancer* **70**: 363–370
Junor EJ, Hole DJ, McNulty et al (1999) Specialist gynaecologists and survival outcome in ovarian cancer: a Scottish study of 1866 patients. *Br J Obstet Gynaecol* **106**: 1130–1136
Kehoe S, Powell J, Wilson S et al (1994) The influence of the operating surgeon’s specialisation on patient survival in ovarian carcinoma. *Br J Cancer* **70**: 1014–1017
Marsoni S, Torri V, Valsecchi MG et al (1990) Prognostic factors in advanced epithelial ovarian cancer. *Br J Cancer* **62**: 444–450
Nguyen NH, Averette HE, Hoskins W et al (1993) National survey of ovarian carcinoma IV. Critical assessment of current International Federation of Obstetrics and Gynaecology Staging System. *Cancer* **72**: 3007–3011
NHS Executive, July 1999. Improving Outcomes in Gynaecological Cancers: Guidance on Commissioning Cancer Services.
Regional Cancer Organisation, April 1999. Management of Gynaecological Carcinoma in the South West of England. South & West.
Trimbos JB, Hellebrekers BWJ, Kenter GG, Peters LAW and Zwinderman KH (2000) The long learning curve of gynaecological cancer surgery: an argument for centralisation. *BJOG* **107**: 19–22

**APPENDIX 1** Contributing hospitals

| | | Cancer Centre 1997 | Hospital |
|---|---|---|---|
| NHS | 1 | Plymouth | Derriford Plymouth |
| | 2 | Plymouth | Treliske RCH Truro |
| | 3 | Exeter | Exeter RD & E |
| | 4 | Exeter | NDDH Barnstable |
| | 5 | Exeter | Torbay |
| | 6 | 3 Counties | CGH Cheltenham |
| | 7 | 3 Counties | GRH Gloucester |
| | 8 | 3 Counties | Hereford County Hospital |
| | 9 | Avon & Somerset | Bath RUH |
| | 10 | Avon & Somerset | Frenchay Bristol |
| | 11 | Avon & Somerset | MPH Taunton |
| | 12 | Avon & Somerset | Southmead Bristol |
| | 13 | Avon & Somerset | St Michael’s Bristol |
| | 14 | Avon & Somerset | Weston General |
| | 15 | Avon & Somerset | Yeovil District Hospital |
| | 16 | Wiltshire | Salisbury (Odstock) |
| | 17 | Wiltshire | Swindon Princess Margaret |
| | 18 | Dorset | Poole Hospital |
| | 19 | Dorset | Royal Bournemouth General |
| | 20 | Dorset | West Dorset Dorchester |
| PRIVATE | | | |
| | 1 | Plymouth | Duchy Cornwall |
| | 2 | Exeter | Mount Stuart |
| | 3 | 3 Counties | Cotswold Nuffield Royal |
| | 4 | 3 Counties | Winfield |
| | 5 | Avon & Somerset | Bath Clinic |
| | 6 | Avon & Somerset | Chesterfield |
| | 7 | Avon & Somerset | Glen Bristol |
| | 8 | Avon & Somerset | Somerset Nuffield |

© 2001 Cancer Research Campaign

**APPENDIX 2** RCO Guidance Notes for Surgeons & Pathologists

### SOUTH AND WEST REGIONAL CANCER ORGANISATION

**King Square House, King Square, Bristol BS2 8EE**

### HISTOPATHOLOGY GUIDELINES

### 1. Cervical carcinoma a) *Squamous carcinoma*—this category includes typical squamous carcinomas showing all grades of differentiation and of both large and small cell subtypes. The category also includes papillary squamous carcinomas and condylomatous carcinomas. Micro-invasive carcinomas of squamous type should also be recorded in this category. Verrucous carcinomas (which are invariably well-differentiated) are also of squamous type and should be included in this category. However, since the behaviour of these tumours differs from the other types of squamous carcinoma, in addition to ticking the "squamous" box on the form, please write "verrucous" alongside the box as well.

b) *Adenocarcinoma*—adenocarcinomas of endocervical, endometrial papillary serous, clear cell and enteric types are all included in this category as is minimal deviation adenocarcinoma. Early invasive adenocarcinoma should also be recorded.

c) *Other*—adenosquamous carcinomas, mixed tumours, small cell carcinomas (basaloid, neuroendocrine), undifferentiated carcinoma, glassy cell carcinoma, adenoid cystic carcinoma and malignant mixed mesodermal (Mullerian) tumours.

### 2. Endometrial carcinoma a) *Usual type*—endometrioid adenocarcinomas are deemed to be of "usual type". This category includes endometrioid adenocarcinomas with squamous/morular metaplasia, secretory carcinomas, papillary and ciliated cell variants.

b) *High grade type*—this category includes papillary serous adenocarcinomas, clear cell, adenosquamous, squamous and undifferentiated carcinomas.

c) *Other*—malignant mixed mesodermal/Mullerian tumours and any other tumours not included in categories a) or b) above.

### 3. Ovarian carcinoma a) *Borderline and invasive epithelial tumours*—include serous, mucinous (endocervical and enteric), transitional/Brenner, endometrioid and clear cell/mesonephroid tumours. Undifferentiated carcinomas are also included in the invasive epithelial group.

b) *Other*—this category includes all other ovarian tumours (including carcinomas arising in teratomas).

### 4. Vulval carcinoma a) *Squamous carcinomas*—include warty/condylomatous carcinoma, spindle cell squamous carcinomas, scams carcinoma with tumor giant cells and acantholytic squamous cell carcinoma.

b) *Verrucous carcinoma*—is listed separately because it differs in clinical behaviour from typical squamous carcinoma.

c) *Other*—basal cell carcinoma, mixed tumours, Paget's disease, Melanomas, adenocarcinomas arising locally within adnexal or vestibular glands and any type of carcinoma no listed in a) or b) above.

*© 2001 Cancer Research Campaign*
